# Biota from the coastal wetlands of Praia da Vitória (Terceira, Azores, Portugal): Part 4 – Vascular plants

**DOI:** 10.3897/BDJ.7.e38687

**Published:** 2019-10-18

**Authors:** Rui B. Elias, Mariana R. Brito, César M.M. Pimentel, Elisabete C. Nogueira, Paulo A. Borges

**Affiliations:** 1 CE3C – Centre for Ecology, Evolution and Environmental Changes/Azorean Biodiversity Group and Universidade dos Açores - Faculdade de Ciências Agrárias e do Ambiente, Angra do Heroísmo, Portugal CE3C – Centre for Ecology, Evolution and Environmental Changes/Azorean Biodiversity Group and Universidade dos Açores - Faculdade de Ciências Agrárias e do Ambiente Angra do Heroísmo Portugal; 2 LIFE CWR – LIFE project “Ecological Restoration and Conservation of Praia da Vitória Coastal Wet Green Infrastructures”, Praia da Vitória, Portugal LIFE CWR – LIFE project “Ecological Restoration and Conservation of Praia da Vitória Coastal Wet Green Infrastructures” Praia da Vitória Portugal

**Keywords:** Azores, Magnoliophyta, Magnoliopsida, Liliopsida, Terceira, Plantae, Pteridophyta, wetlands

## Abstract

**Background:**

The data presented here come from field observations, carried out between 2014 and 2017, as part of a LIFE research project aiming to preserve and restore three coastal wetlands of Praia da Vitória (Terceira Island, Azores, Portugal) (LIFE-CWR). A total of 23 vascular plant species surveys were carried out in three sites: one for each semester in Paul da Praia da Vitória (PPV) and Paul da Pedreira do Cabo da Praia (PPCP); one for each semester (except in 2014) in Paul do Belo Jardim (PBJ). The main objectives were to determine the plant richness of the three sites and to monitor yearly variation on species composition.

**New information:**

A total of 107 taxa, belonging to 50 families, were observed, many of which are new records for the area, especially in PBJ and PPCP, where 78 and 92% of species records were new. A few very rare species in the Azores were recorded in these coastal wetlands, namely *Lotus creticus*, *Bolboschoenus maritimus*, *Juncus maritimus* and *Polygonum maritimum*.

## Introduction

Before human settlement in the 15th century, Azorean natural vegetation was most probably dominated by forests (Elias et al. 2016). Those forest habitats were gradually replaced (except for a few mountainous areas of some islands) by agriculture fields, urban areas, pastures, production forests and exotic forests (Martins 1993). In coastal areas, natural vegetation (where it still remains) consists essentially of supratidal communities (with the endemics *Azorina vidalii*, *Euphorbia azorica* and *Spergularia azorica*), coastal grasslands (mainly of *Festuca petraea*) and coastal scrublands, usually dominated by *Erica azorica* and *Morella faya*. Given that rocky cliffs dominate the Azorean coastline and human-induced habitat changes were higher on low elevation areas, dune communities and coastal wetlands are extremely rare. Coastal wetlands usually have shallow lagoons, separated from the sea by natural barriers. Typically, lagoons are fringed by mangroves in the tropics and marsh plants, like *Juncus*, in the temperate zones (Morton 2014).

In the Azores, only six sites can be properly called coastal wetlands: Lajes do Pico (Pico Island), Fajã do Santo Cristo and Fajã dos Cubres (in São Jorge Island) and the three sites of the coastal wetland complex of Praia da Vitória (Terceira Island). These habitats are home to rare species (in the Azores), like the aquatic plants *Ruppia maritima* and *R. spiralis* (Morton 2019). The coastal wetland complex of Praia da Vitória (Terceira Island, Azores, Portugal) is composed by Paul da Praia da Vitória (PPV), Paul do Belo Jardim (PBJ) and Paul da Pedreira do Cabo da Praia (PPCP). Since the earlier works by Dias et al. (1991), Morton et al. (1997) and Morton et al. (1998), the first comprehensive study of Praia da Vitória wetlands was done by the LIFE-CWR coastal wetlands restoration project, under the responsibility of Praia da Vitória Municipality. The present data paper is the fourth of a series dealing with the biota from these coastal wetlands (see Borges et al. 2018, Gabriel et al. 2019, Goulart et al. 2019).

## General description

### Purpose

This work is part of a comprehensive study regarding the biodiversity of the coastal wetlands of Praia da Vitória (Terceira, Azores) under the scope of the LIFE-CWR coastal wetlands restoration project. The aim of this work was to inventory the vascular plants present in the three coastal areas of Praia da Vitória (PPV, PBJ and PPCP), in order to determine the plant richness of the three sites and the yearly variation in species composition.

## Project description

### Title

Inventory of vascular plant species of three coastal wetlands of Terceira Island (Azores)

### Personnel

The inventory was conducted during four years between April 2014 and September 2017 by Mariana R. Brito, with the collaboration of César Pimentel, under the responsibility of Elisabete C. Nogueira and advice of Rui B. Elias. Species identification was performed by Mariana R. Brito and Rui B. Elias. Paulo A. V. Borges coordinated the publication of the series of data papers regarding the biodiversity of Terceira island coastal wetlands (arthropods, bryophytes, vascular plants and birds).

### Study area description

**Figure 1.**
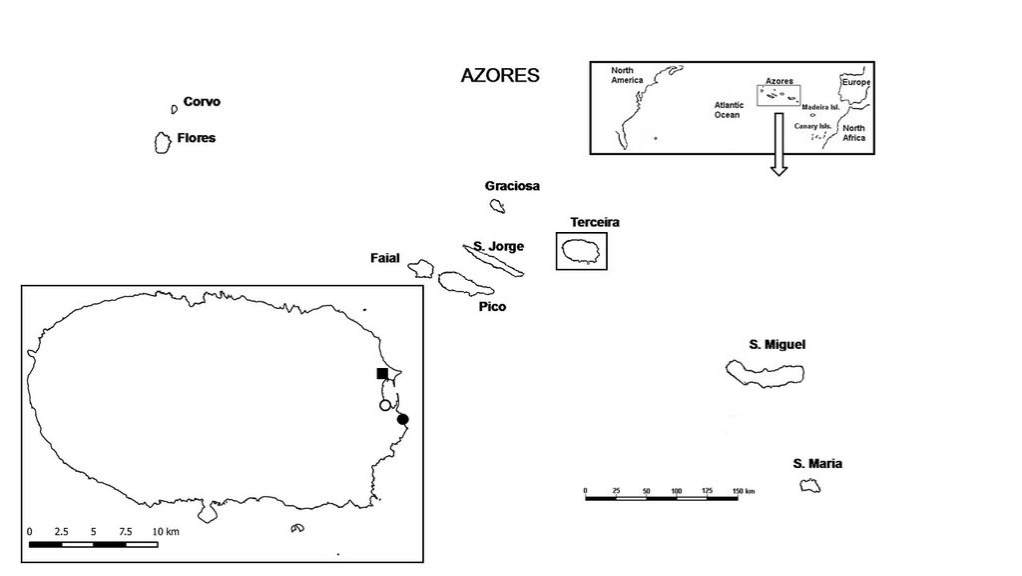
Location of the study areas in Terceira island: ■ Paul da Praia da Vitória (PPV); ○ Paul do Belo Jardim (PBJ); ● Paul da Pedreira do Cabo da Praia (PPCP). The geographical setting of the Azores islands and the location of the archipelago in the North Atlantic Ocean are also shown.

The Azores belong to the Holarctic Biogeographical Kingdom and Eurosiberian Region (Rivas-Martinez et al. 2002). Terceira Island is the third largest island of the archipelago and has the fourth longest shoreline (Forjaz et al. 2004) (Fig. 1). Like other Azorean islands (with the exception of Santa Maria and Graciosa), the prevalent type of climate in Terceira is temperate with no dry season and with a mild summer (Köppen Climate Classification - Cfb). However, in Praia da Vitória (a lowland area in the east of the island), the climate is temperate with hot and dry summers (Csa) (Atlas 2012). For more details on the description of the study area see Borges et al. (2018). During the study period, conservation measures were implemented, namely the creation/enlargement of water bodies in PBJ and PPV and the improvement of bird watching regulation and control of *Arundo donax* in PPCP and PPV.

### Design description

A total of 23 surveys were carried out in three sites (PPV, PBJ and PPCP). Surveys took place in each semester (except for PBJ in 2014) during 2-3 days (depending on the dimension of the study areas), for a total of 51 days of direct observation. Study areas were delimited using GPS and included the margins of water bodies and the surrounding terrestrial areas. 

### Funding

LIFE CWR – Ecological Restoration and Conservation of Praia da Vitória Coastal Wet Green Infrastructure (2013-2018) funded the field work. AZORESBIOPORTAL – PORBIOTA (ACORES-01-0145-FEDER-000072) funded the open access of biodiversity data.

## Sampling methods

### Study extent

This study covers a small coastal area with 3.58 km extension between PPV and PPCP.

**Study dates:** April 2014 – September 2017

### Sampling description

In each survey, the presence of vascular plant taxa was recorded. For the most common taxa, plant samples were collected, dried and stored in the Environment Division of Praia da Vitória Municipality. Most taxa were identified in the field. Whenever this was not possible, a plant sample was collected and the identification confirmed later. A photo archive of the recorded taxa was also done. Information and taxonomical keys from Franco and Afonso (1994), Franco and Afonso (1998), Franco and Afonso (2003) and Schäfer (2005) were used for taxon identification. Nomenclature follows Silva et al. (2010). 

## Geographic coverage

### Description

Terceira Island (Azores), Macaronesia, Portugal

### Coordinates

38°42’09’’N and 38°42’47.95’N Latitude; 27°02’39’’ and 27°03’46’’ Longitude.

## Taxonomic coverage

### Description

Plantae - Spermatophyta

## Temporal coverage

### Notes

April 2014 – September 2017

## Usage rights

### Use license

Open Data Commons Attribution License

## Data resources

### Data package title

LIFE_CWR_TER_Plants

### Resource link

http://ipt.gbif.pt/ipt/resource?r=azorean_vascularplants

### Alternative identifiers

http://islandlab.uac.pt/software/ver.php?id=33

### Number of data sets

1

### Data set 1.

#### Data set name

Vascular Plants from Praia da Vitória

#### Data format

Darwin Core Archive

#### Number of columns

51

#### Download URL

http://ipt.gbif.pt/ipt/resource?r=azorean_vascularplants

#### Data format version

version 1

#### Description

In this data table, we include all the records for which a taxonomic identification of the species was possible. The dataset submitted to GBIF (Global Biodiversity Information Facility) is structured as a sample event dataset, with two tables: event (as core) and occurrences. The data in this sampling event resource have been published as a Darwin Core Archive (DwCA), which is a standardised format for sharing biodiversity data as a set of one or more data tables. The core data table contains 23 records. One extension data table also exists. An extension record supplies extra information about a core record. The number of records in each extension data table is illustrated in the IPT (Integrated Publishing Toolkit) link. This IPT archives the data and thus serves as the data repository. The data and resource metadata are available for downloading in the downloads section. The versions table lists other versions of the resource that have been made publicly available and allows tracking changes made to the resource over time.

**Data set 1.** 

Column label	Column description
Table Events	The sub-table with events
country	Country of the sampling site
countryCode	ISO code of the country of the sampling site
stateProvince	Name of the region of the sampling site
islandGroup	Name of archipelago
Island	Name of the island
municipality	Name of the municipality
locationRemarks	Details on the locality site
eventID	Identifier of the events, unique for the dataset
fieldNumber	Number given to each sample
verbatimCoordinates	Original coordinates recorded
decimalLatitude	Approximate centre point decimal latitude of the field site in GPS coordinates
decimalLongitude	Approximate centre point decimal longitude of the field site in GPS coordinates
coordinatePrecision	Precision of the coordinates
geodeticDatum	The reference point for the various coordinate systems used in mapping the earth
georeferenceSources	Method used to obtain coordinates
minimumElevationInMetres	Minimum elevation in metres
maximumElevationInMetres	Maximum elevation in metres
eventDate	Date or date range the sampling
startDayOfYear	Day of the year the sampling started
endDayOfYear	Day of the year the sampling ended
samplingProtocol	The sampling protocol used to capture the species
samplingEffort	The amount of time of each sampling
sampleSizeValue	The numeric amount of time spent in each sampling
sampleSizeUnit	The unit of the sample size value
taxonRank	Taxonomic rank to which the specimens were identified
Table Occurrences	The sub-table with occurrence data
Type	Type of the record, as defined by the Public Core standard
occurrenceID	Identifier of the record, coded as a global unique identifier
licence	Reference to the licence under which the record is published
InstitutionCode	The code of the institution publishing the data
InstitutionID	The identity of the institution publishing the data
datasetName	Name of the dataset
basisOfRecord	The nature of the data record
recordedBy	Name of the person who performed the sampling of the specimens
eventID	Identifier of the events, unique for the dataset
recordedBy	Name of the person who performed the sampling of the specimens
kingdom	Kingdom name
phylum	Phylum name
class	Class name
order	Order name
family	Family name
genus	Genus name
specificEpithet	Specific epithet
infraspecificEpithet	Infraspecific epithet, when available
scientificNameAuthorship	Name of the author of the lowest taxon rank included in the record
scientificName	Complete scientific name including author and year
taxonRank	Lowest taxonomic rank of the record
establishmentMeans	The process of establishment of the species in the location, using a controlled vocabulary: 'native non-endemic', 'introduced', 'endemic'.
identifiedBy	Name of the person who made the identification
dateIdentified	Date on which the record was identified

## Additional information

During the four-year observation period (2014-2017), a total of 107 taxa, belonging to 50 families, were observed (Table 1). Almost all were flowering plants (only three fern species were recorded), mostly Magnoliopsida (75%). Regarding the colonisation status, 83% were introduced species and only 17% were native non-endemic or endemic. The number of species in the three sites ranged from 74, in PPCP, to 79, in PPV. In this contribution, we add 23 records for PPV, 61 records for PBJ and 68 records for PPCP.

The percentage of native species ranged between 15% in PPV and 18% in PBJ. These coastal habitats are surrounded by urban areas and pastures and the high percentage of exotic species is the natural consequence of the degree of disturbance that these wetlands have experienced in the past and the human pressure that they still endure.

**Figure 2.**
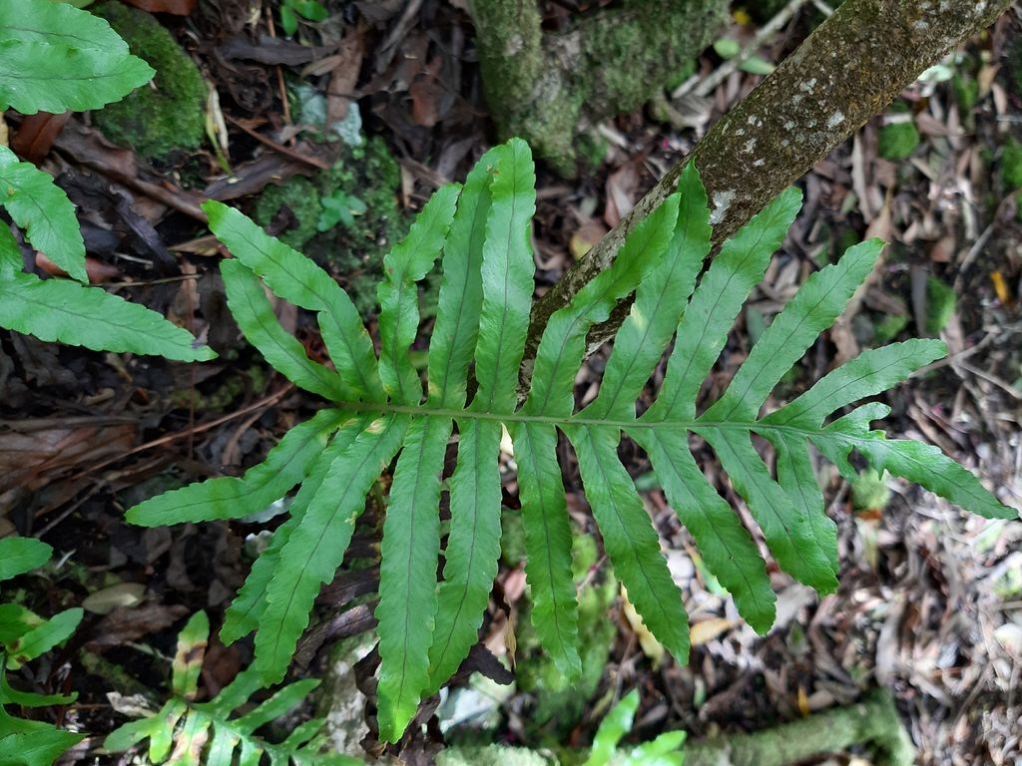
*Polypodium azoricum* (Photo by Rui Elias).

**Figure 3.**
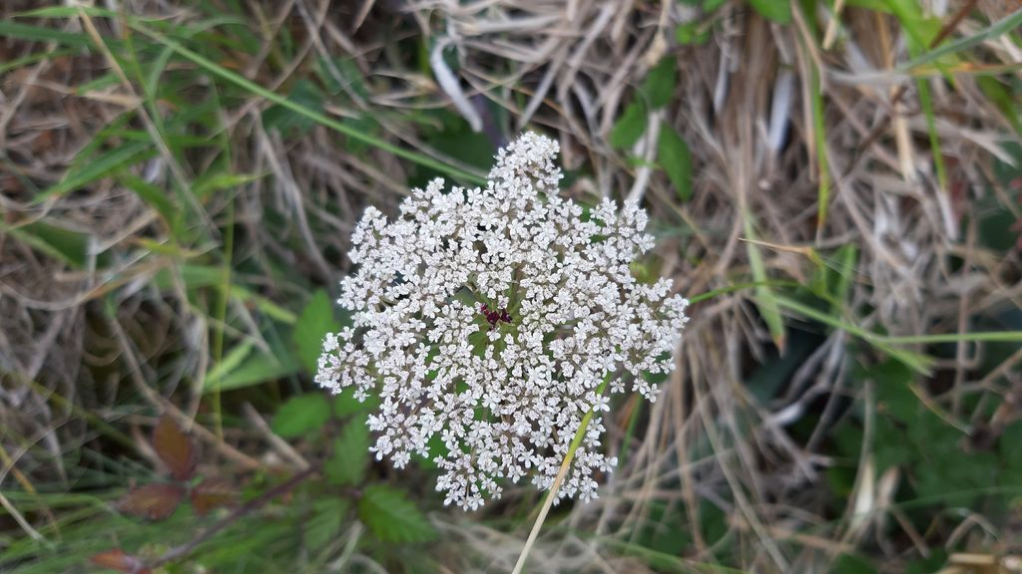
Daucus carota subsp. azoricus (Photo by Rui Elias).

**Figure 4.**
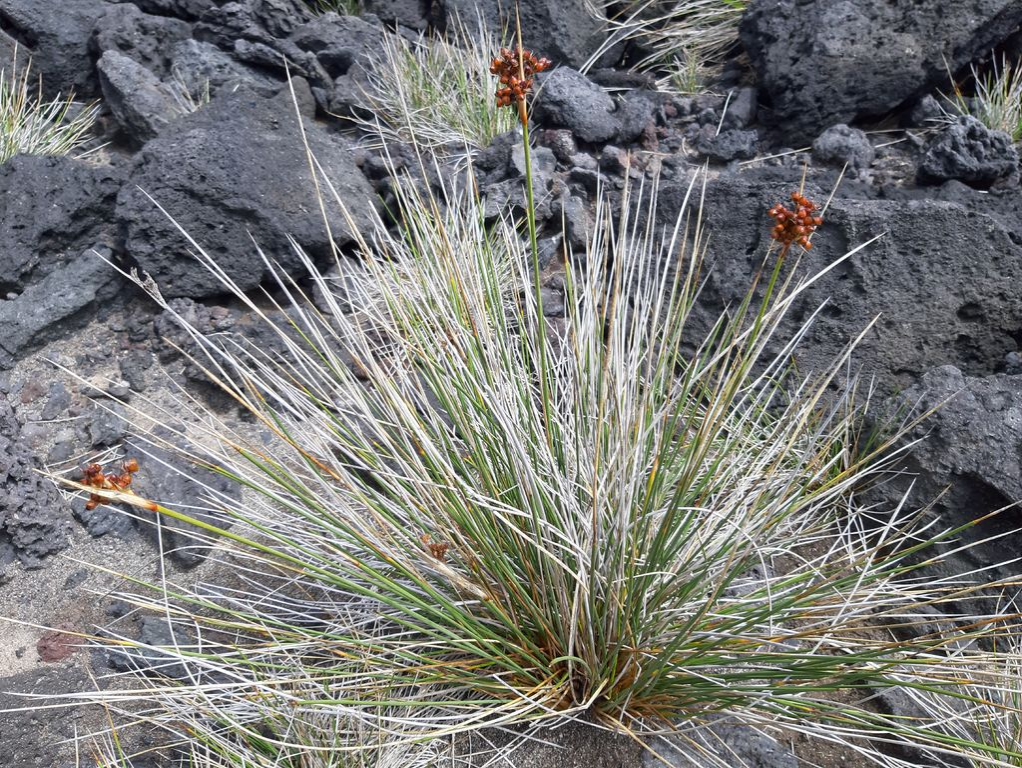
*Juncus acutus* (Photo by Rui Elias).

**Figure 5.**
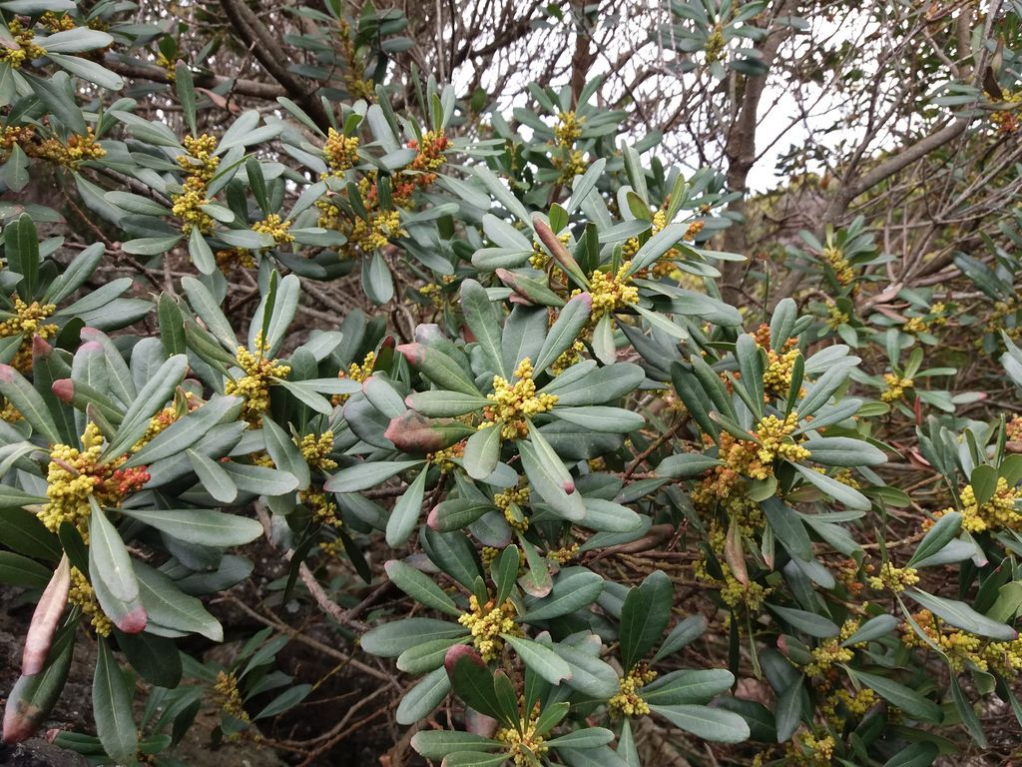
*Morella faya* (Photo by Rui Elias).

Overal, only 18 native (endemic and non-endemic) taxa were present in these wetlands. *Polypodium azoricum* (Fig. 2), *Laurus azorica* and *Daucus carota* subsp. *azoricus*(Fig. 3) were the only endemic taxa recorded. The latter is typical of coastal areas, but the others (especially *L. azorica*) are not commonly found at such low altitudes. Amongst native non-endemic species, *Juncus acutus* (Fig. 4) and *Morella faya* (Fig. 5) are diagnostic species of Azorean natural supratidal communities and coastal scrublands, respectively.

**Figure 6.**
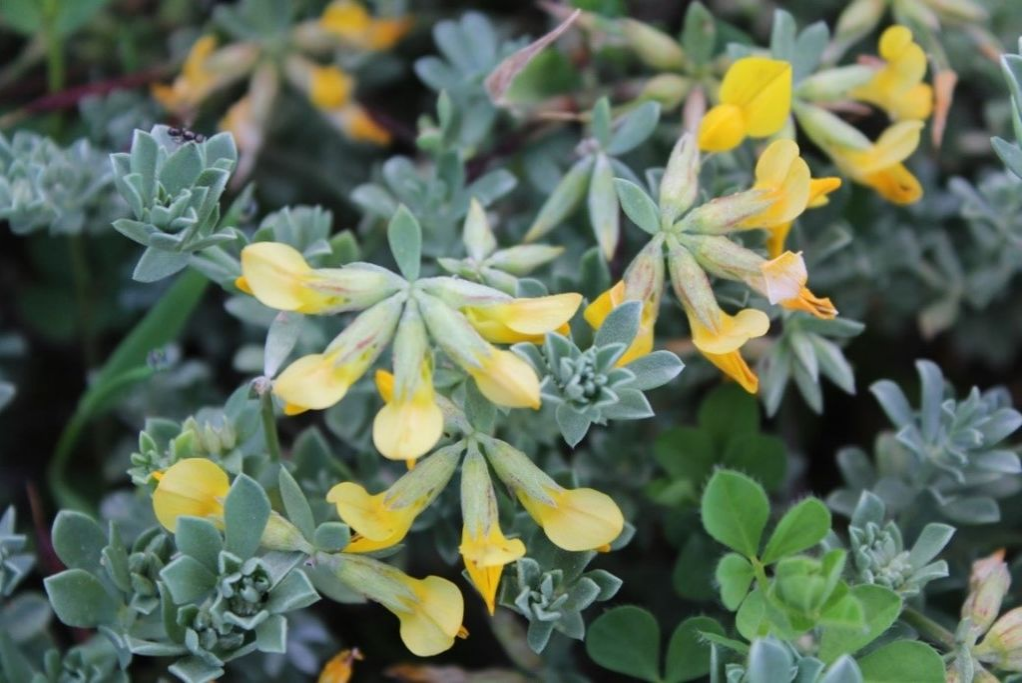
*Lotus creticus* (Photo by LIFE-CWR).

**Figure 7.**
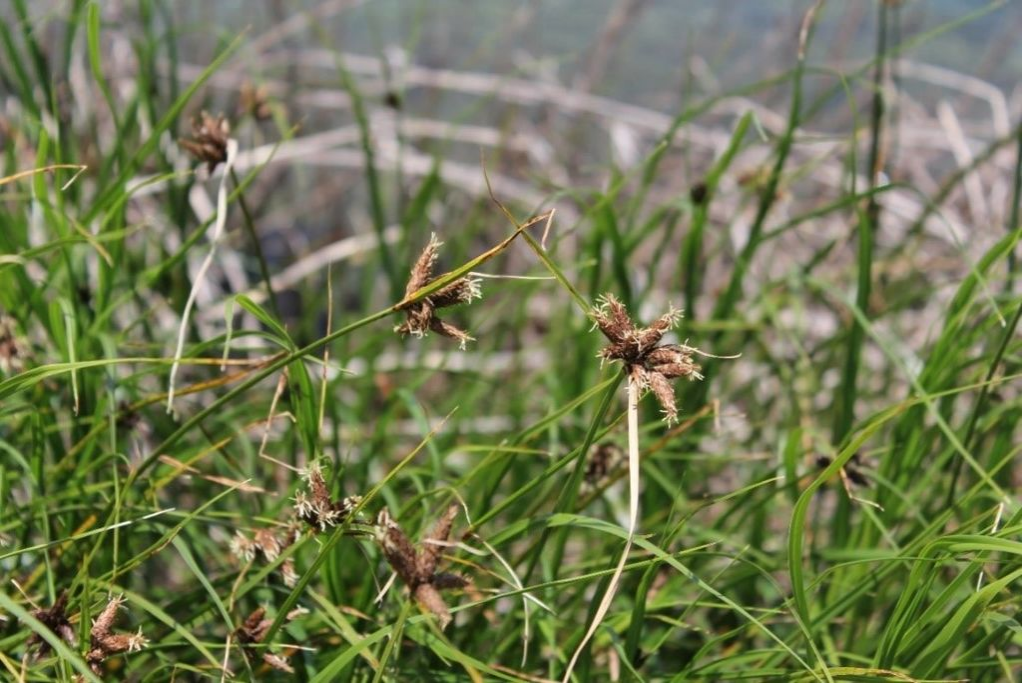
*Bolboschoenus maritimus* (Photo by LIFE-CWR).

**Figure 8.**
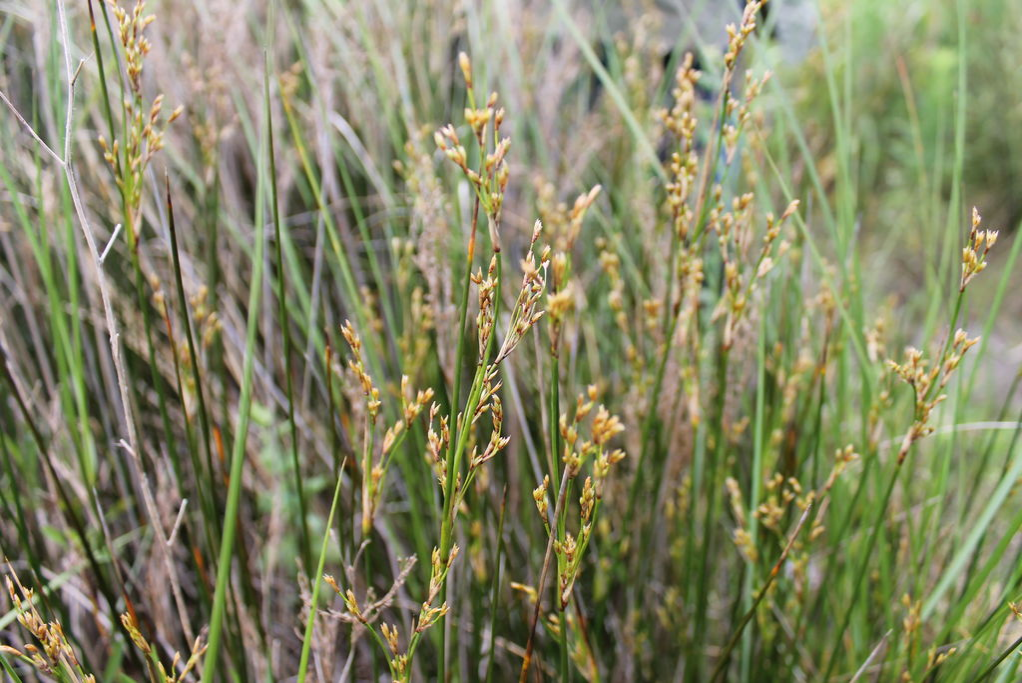
*Juncus maritimus* (Photo by Rui Elias).

A few rare species, in the Azores, were also found, namely *Lotus creticus*, *Bolboschoenus maritimus*, *Juncus maritimus* and *Polygonum maritimum*. *Lotus creticus* (Fig. 6) and *Bolboschoenus maritimus* (Fig. 7) occur only in Terceira. *Juncus maritimus* (Fig. 8) occurs in a few coastal areas of the islands of Pico, São Jorge and Terceira. *Polygonum maritimum*, is an equally rare species that can be found only in São Miguel, Terceira, Faial and Pico (Silva et al. 2010). All these species are threatened because of the low number of surviving populations (and individuals), habitat change and human pressure.

Variation in species composition was higher in PPV and PPCP but only significant in the latter (Qui-square 43.6; p < 0.05). In PPCP, 39 taxa were recorded in 2014, 59 in 2015 and 2016 and 69 in 2017. These differences may be, at least in part, attributed to the conservation measures applied in this wetland, namelly the increased regulation of bird-watching activities and control of the invasive species *Arundo donax*.

### Concluding remarks

This is the fourth contribution, based on a comprehensive project that aimed to inventory the biota of a rare habitat in the Azores (coastal wetland). In previous contributions, arthropods (Borges et al. 2018), bryophytes (Gabriel et al. 2019) and birds (Goulart et al. 2019) were listed with taxonomical and ecological remarks. Amongst those records, 11 were new for the Azores and 19 were new for Terceira. Overall, during this project, 489 taxa were recorded: 58 bryophytes, 107 vascular plants, 216 arthropods and 108 birds. As expected, because these wetlands were subjected to severe anthropogenic disturbances, for arthropods and vascular plants, most taxa are exotic. Nevertheless, for both groups, a few rare species were found. This series of papers has demonstrated the importance of the coastal wetlands of Praia da Vitória in the Azorean context. Active conservation and ecological restoration must continue to be a priority for the stakeholders.

## Figures and Tables

**Table 1.** List of vascular plants observed in the three coastal wetlands of Praia da Vitória (Terceira Island, Azores, Portugal) - *Paul da Praia da Vitória* (PPV), *Paul do Belo Jardim* (PBJ) and *Paul da Pedreira do Cabo da Praia* (PPCP). For each taxon, Phylum, Class, Order and Family are indicated. Colonisation status follows Silva et al. (2010): Introduced (INT), Native (NAT) or Endemic (END).

**Phylum**	**Class**	**Order**	**Family**	**Taxon**	**Colonization**	**PPV**	**PBJ**	**PPCP**
Pteridophyta	Polypodiopsida	Polypodiales	Dryopteridaceae	*Cyrtomium falcatum* (L.fil.) C. Presl	INT			x
Pteridophyta	Polypodiopsida	Polypodiales	Polypodiaceae	*Polypodium azoricum* (Vasc) R. Fern.	END			x
Pteridophyta	Polypodiopsida	Polypodiales	Dennstaedtiaceae	*Pteridium aquilinum* (L.) Kuhn	NAT	x	x	x
Magnoliophyta	Magnoliopsida	Laurales	Lauraceae	*Laurus azorica* (Seub.) Franco	END	x		x
Magnoliophyta	Magnoliopsida	Ranunculales	Papaveraceae	Fumaria muralis Sonder ex Koch subsp. muralis	INT	x	x	x
Magnoliophyta	Magnoliopsida	Ranunculales	Papaveraceae	*Papaver dubium* L.	INT	x		
Magnoliophyta	Magnoliopsida	Ranunculales	Papaveraceae	*Papaver rhoeas* L.	INT		x	x
Magnoliophyta	Magnoliopsida	Ranunculales	Ranunculaceae	*Ranunculus repens* L.	INT	x	x	
Magnoliophyta	Magnoliopsida	Proteales	Proteaceae	*Banksia integrifolia* L.	INT	x		
Magnoliophyta	Magnoliopsida	Fagales	Myricaceae	*Morella faya* (Aiton) Wilbur	NAT	x		x
Magnoliophyta	Magnoliopsida	Rosales	Rosaceae	*Rubus ulmifolius* Schott	INT	x	x	x
Magnoliophyta	Magnoliopsida	Rosales	Urticaceae	*Urtica membranacea* Poir.	INT		x	x
Magnoliophyta	Magnoliopsida	Fabales	Fabaceae	*Lotus creticus* L.	NAT	x	x	x
Magnoliophyta	Magnoliopsida	Fabales	Fabaceae	*Lotus parviflorus* Desf.	INT	x		x
Magnoliophyta	Magnoliopsida	Fabales	Fabaceae	*Medicago lupulina* L.	INT	x	x	x
Magnoliophyta	Magnoliopsida	Fabales	Fabaceae	*Melilotus indicus* (L.) All.	INT	x	x	
Magnoliophyta	Magnoliopsida	Fabales	Fabaceae	*Trifolium fragiferum* L.	INT	x		
Magnoliophyta	Magnoliopsida	Fabales	Fabaceae	*Trifolium pratense* L.	INT	x	x	
Magnoliophyta	Magnoliopsida	Fabales	Fabaceae	*Trifolium repens* L.	INT	x	x	x
Magnoliophyta	Magnoliopsida	Fabales	Fabaceae	*Vicia sativa* L. subsp. sativa	INT	x	x	x
Magnoliophyta	Magnoliopsida	Oxalidales	Oxalidaceae	*Oxalis pes-caprae* L.	INT	x	x	
Magnoliophyta	Magnoliopsida	Malpighiales	Euphorbiaceae	*Ricinus communis* L.	INT	x	x	x
Magnoliophyta	Magnoliopsida	Malvales	Malvaceae	*Malva pseudolavatera* Webb & Berthel.	INT	x	x	x
Magnoliophyta	Magnoliopsida	Malvales	Malvaceae	*Sida rhombifolia* L.	INT	x	x	x
Magnoliophyta	Magnoliopsida	Brassicales	Brassicaceae	*Lobularia maritima* (L.) Desv.	INT		x	x
Magnoliophyta	Magnoliopsida	Brassicales	Brassicaceae	Raphanus raphanistrum L. subsp. raphanistrum	INT		x	
Magnoliophyta	Magnoliopsida	Brassicales	Brassicaceae	Rapistrum rugosum (L.) All. subsp. rugosum	INT			x
Magnoliophyta	Magnoliopsida	Brassicales	Resedaceae	*Reseda luteola* L.	INT	x	x	x
Magnoliophyta	Magnoliopsida	Brassicales	Tropaeolaceae	*Tropaeolum majus* L.	INT	x	x	x
Magnoliophyta	Magnoliopsida	Myrtales	Onagraceae	*Oenothera rosea* L'Hér. ex Aiton	INT	x	x	x
Magnoliophyta	Magnoliopsida	Geraniales	Geraniaceae	*Geranium dissectum* L.	INT		x	
Magnoliophyta	Magnoliopsida	Geraniales	Geraniaceae	*Geranium molle* L.	INT		x	
Magnoliophyta	Magnoliopsida	Geraniales	Geraniaceae	*Geranium purpureum* Vill.	INT	x	x	x
Magnoliophyta	Magnoliopsida	Saxifragales	Crassulaceae	*Umbilicus rupestris* (Salisb.) Dandy	INT			x
Magnoliophyta	Magnoliopsida	Caryophyllales	Aizoaceae	*Tetragonia tetragonoides* (Pall.) O. Kuntze	INT		x	x
Magnoliophyta	Magnoliopsida	Caryophyllales	Amaranthaceae	*Atriplex prostrata* Boucher ex DC.	NAT	x	x	x
Magnoliophyta	Magnoliopsida	Caryophyllales	Amaranthaceae	Salsola kali L. subsp. tragus (L.) Nyman	INT		x	
Magnoliophyta	Magnoliopsida	Caryophyllales	Caryophyllaceae	*Silene gallica* L.	INT	x		x
Magnoliophyta	Magnoliopsida	Caryophyllales	Caryophyllaceae	*Spergularia marina* (L.) Griseb	INT			x
Magnoliophyta	Magnoliopsida	Caryophyllales	Phytolaccaceae	*Phytolacca americana* L.	INT	x	x	x
Magnoliophyta	Magnoliopsida	Caryophyllales	Polygonaceae	*Persicaria capitata* (Buch. Ham. ex D. Don) H. Gross	INT			x
Magnoliophyta	Magnoliopsida	Caryophyllales	Polygonaceae	*Polygonum maritimum* L.	NAT		x	
Magnoliophyta	Magnoliopsida	Caryophyllales	Portulaceae	Portulaca oleracea L. subsp. oleraceae	INT	x	x	x
Magnoliophyta	Magnoliopsida	Caryophyllales	Tamaricaceae	*Tamarix africana* Poir.	INT	x	x	
Magnoliophyta	Magnoliopsida	Ericales	Primulaceae	*Anagallis arvensis* L.	INT	x	x	x
Magnoliophyta	Magnoliopsida	Gentianales	Gentianaceae	*Centaurium scilloides* (L. Fil.) Samp.	NAT		x	
Magnoliophyta	Magnoliopsida	Gentianales	Rubiaceae	*Galium aparine* L.	INT	x	x	
Magnoliophyta	Magnoliopsida	Gentianales	Rubiaceae	*Sherardia arvensis* L.	INT	x	x	x
Magnoliophyta	Magnoliopsida	Lamiales	Lamiaceae	*Clinopodium ascendens* (Jord.) Samp.	NAT		x	x
Magnoliophyta	Magnoliopsida	Lamiales	Lamiaceae	*Mentha pulegium* L.	NAT		x	
Magnoliophyta	Magnoliopsida	Lamiales	Lamiaceae	*Mentha suaveolens* Ehrh.	INT	x	x	x
Magnoliophyta	Magnoliopsida	Lamiales	Orobanchaceae	*Orobanche minor* Sm.	INT	x		
Magnoliophyta	Magnoliopsida	Lamiales	Orobanchaceae	*Parentucellia viscosa* (L.) Caruel	INT		x	x
Magnoliophyta	Magnoliopsida	Lamiales	Plantaginaceae	*Plantago coronopus* L.	NAT	x	x	x
Magnoliophyta	Magnoliopsida	Lamiales	Plantaginaceae	*Plantago lanceolata* L.	INT	x	x	x
Magnoliophyta	Magnoliopsida	Lamiales	Plantaginaceae	*Plantago major* L.	INT	x		x
Magnoliophyta	Magnoliopsida	Lamiales	Scrophulariaceae	*Veronica persica* Poir.	INT	x		
Magnoliophyta	Magnoliopsida	Lamiales	Verbenaceae	*Lantana camara* L.	INT	x	x	x
Magnoliophyta	Magnoliopsida	Lamiales	Verbenaceae	*Verbena bonariensis* L.	INT	x	x	
Magnoliophyta	Magnoliopsida	Lamiales	Verbenaceae	*Verbena officinalis* L.	INT	x	x	x
Magnoliophyta	Magnoliopsida	Lamiales	Verbenaceae	*Verbena rigida* Spreng.	INT	x		
Magnoliophyta	Magnoliopsida	Solanales	Convolvulaceae	Convolvulus arvensis L. subsp. arvensis	INT		x	x
Magnoliophyta	Magnoliopsida	Solanales	Convolvulaceae	*Ipomoea indica* (Burm.fil.) Merr.	INT	x		
Magnoliophyta	Magnoliopsida	Solanales	Solanaceae	*Datura stramonium* L.	INT	x	x	x
Magnoliophyta	Magnoliopsida	Solanales	Solanaceae	*Physalis peruviana* L.	INT	x		x
Magnoliophyta	Magnoliopsida	Solanales	Solanaceae	*Salpichroa origanifolia* (Lam.) Baill.	INT	x	x	x
Magnoliophyta	Magnoliopsida	Solanales	Solanaceae	*Solanum nigrum* L.	INT	x	x	x
Magnoliophyta	Magnoliopsida	Boraginales	Boraginaceae	*Echium plantagineum* L.	INT		x	x
Magnoliophyta	Magnoliopsida	Asterales	Asteraceae	*Ageratina adenophora* (Spreng.) R. M. King & H. Rob.	INT	x		x
Magnoliophyta	Magnoliopsida	Asterales	Asteraceae	*Conyza bonariensis* (L.) Cronquist	INT	x	x	x
Magnoliophyta	Magnoliopsida	Asterales	Asteraceae	*Cichorium intybus* L.	INT	x	x	x
Magnoliophyta	Magnoliopsida	Asterales	Asteraceae	*Dittrichia viscosa* (L.) Greuter	INT			x
Magnoliophyta	Magnoliopsida	Asterales	Asteraceae	*Erigeron karvinskianus* DC.	INT	x		x
Magnoliophyta	Magnoliopsida	Asterales	Asteraceae	*Galactites tomentosa* Moench	INT	x	x	x
Magnoliophyta	Magnoliopsida	Asterales	Asteraceae	*Helminthotheca echioides* (L.) Holub	INT	x	x	x
Magnoliophyta	Magnoliopsida	Asterales	Asteraceae	*Hypochaeris radicata* L.	INT	x	x	x
Magnoliophyta	Magnoliopsida	Asterales	Asteraceae	*Leontodon saxatilis* Lam. susp. *longirostris* (Finch & P. D. Sell) P. Silva	INT	x	x	x
Magnoliophyta	Magnoliopsida	Asterales	Asteraceae	*Pseudognaphalium luteoalbum* (L.) Hilliard & B. L. Burtt	NAT	x	x	x
Magnoliophyta	Magnoliopsida	Asterales	Asteraceae	Solidago gigantea Aiton subsp. serotina McNeill	NAT	x		
Magnoliophyta	Magnoliopsida	Asterales	Asteraceae	Sonchus asper (L.) Hill subsp. asper	INT	x	x	x
Magnoliophyta	Magnoliopsida	Apiales	Apiaceae	Daucus carota L. subsp. azoricus Franco	END	x	x	x
Magnoliophyta	Magnoliopsida	Apiales	Apiaceae	*Foeniculum vulgare* Mill.	INT	x	x	x
Magnoliophyta	Magnoliopsida	Apiales	Araliaceae	*Tetrapanax papyriferus* (Hook.) K. Koch	INT	x		
Magnoliophyta	Magnoliopsida	Apiales	Pittosporaceae	*Pittosporum undulatum* Vent.	INT	x	x	x
Magnoliophyta	Liliopsida	Alismatales	Araceae	*Zantedeschia aethiopica* (L.) Spreng.	INT		x	
Magnoliophyta	Liliopsida	Asparagales	Amaryllidaceae	*Brunsvigia rosea* (Lam.) L. S. Hannibal	INT	x		
Magnoliophyta	Liliopsida	Asparagales	Asparagaceae	*Agave americana* L.	INT		x	
Magnoliophyta	Liliopsida	Poales	Cyperaceae	*Bolboschoenus maritimus* (L.) Palla	NAT	x		
Magnoliophyta	Liliopsida	Poales	Cyperaceae	*Cyperus eragrostis* Lam.	INT	x	x	x
Magnoliophyta	Liliopsida	Poales	Cyperaceae	*Cyperus esculentus* L.	INT	x	x	x
Magnoliophyta	Liliopsida	Poales	Cyperaceae	*Cyperus longus* L.	NAT	x	x	x
Magnoliophyta	Liliopsida	Poales	Juncaceae	*Juncus acutus* L.	NAT	x	x	x
Magnoliophyta	Liliopsida	Poales	Juncaceae	*Juncus maritimus* Lam.	NAT		x	
Magnoliophyta	Liliopsida	Poales	Poaceae	*Arundo donax* L.	INT	x	x	x
Magnoliophyta	Liliopsida	Poales	Poaceae	*Briza maxima* L.	INT		x	x
Magnoliophyta	Liliopsida	Poales	Poaceae	*Bromus catharticus* Vahl	INT	x	x	
Magnoliophyta	Liliopsida	Poales	Poaceae	*Cortaderia selloana* (Schult. & Schult. fil.) Asch. & Graebn.	INT	x		
Magnoliophyta	Liliopsida	Poales	Poaceae	*Cynodon dactylon* (L.) Pers.	INT	x	x	x
Magnoliophyta	Liliopsida	Poales	Poaceae	*Dactylis glomerata* L.	INT			x
Magnoliophyta	Liliopsida	Poales	Poaceae	*Holcus lanatus* L.	INT	x	x	x
Magnoliophyta	Liliopsida	Poales	Poaceae	Hordeum murinum L. subsp. leporinum (Link) Asch. & Graebn.	INT	x	x	x
Magnoliophyta	Liliopsida	Poales	Poaceae	*Lagurus ovatus* L.	INT	x	x	x
Magnoliophyta	Liliopsida	Poales	Poaceae	*Paspalum dilatatum* Poir.	INT	x	x	x
Magnoliophyta	Liliopsida	Poales	Poaceae	*Spartina versicolor* Fabre	INT	x	x	
Magnoliophyta	Liliopsida	Poales	Poaceae	*Sporobolus africanus* (Poir.) Robyns & Tournay	INT	x	x	x
Magnoliophyta	Liliopsida	Zingiberales	Cannaceae	*Canna indica* L.	INT	x		
Magnoliophyta	Liliopsida	Commelinales	Commelinaceae	*Tradescantia fluminensis* Vell.	INT		x	
